# Secular Trends in Blood Pressure Among Japanese Schoolchildren: A Population-Based Annual Survey From 1994 to 2010

**DOI:** 10.2188/jea.JE20110137

**Published:** 2012-09-05

**Authors:** Takako Shirasawa, Hirotaka Ochiai, Rimei Nishimura, Aya Morimoto, Naoki Shimada, Tadahiro Ohtsu, Hiromi Hoshino, Naoko Tajima, Akatsuki Kokaze

**Affiliations:** 1 Department of Public Health, Showa University School of Medicine, Tokyo, Japan; 1 昭和大学医学部公衆衛生学部門; 2 Division of Diabetes, Metabolism and Endocrinology, Department of Internal Medicine, Jikei University School of Medicine, Tokyo, Japan; 2 東京慈恵会医科大学糖尿病・代謝・内分泌内科; 3 Jikei University School of Medicine, Tokyo, Japan; 3 東京慈恵会医科大学

**Keywords:** blood pressure, body mass index, schoolchildren, secular trends

## Abstract

**Background:**

Monitoring secular trends in blood pressure (BP) among children is important in predicting subsequent hypertension and cardiovascular disease. We investigated secular trends in BP using data from population-based annual screenings of Japanese schoolchildren.

**Methods:**

The participants were 10 894 children (all fourth graders between 1994 and 2010 and all seventh graders between 1997 and 2010) living in the town of Ina in Saitama Prefecture, Japan. Body height, weight, and BP were measured, after which children were classified as non-overweight, overweight, or obese. Trends in variables relative to calendar year were analyzed using regression models.

**Results:**

Systolic BP was significantly associated with calendar year among fourth- and seventh-grade boys (−0.350 and −0.434 mm Hg/year, respectively) and fourth- and seventh-grade girls (−0.513 and −0.473 mm Hg/year, respectively) (all *P* < 0.001), respectively, over time. Systolic BP and calendar year were significantly negatively correlated regardless of physique or sex among all fourth graders, but not among obese seventh-grade girls. In addition, diastolic BP and calendar year did not significantly correlate among seventh-grade overweight or obese boys or obese seventh-grade girls.

**Conclusions:**

BP decreased among fourth-grade schoolchildren in Ina during the past 17 years, regardless of sex or physique. However, BP and calendar year did not significantly correlate among obese seventh graders.

## Abstract (ja)

小児期の血圧の年次推移を観察することは、将来の高血圧や心血管疾患発症の動向を予測する際に重要である。そこで本研究は、日本人学童・生徒における血圧の年次推移について体格別に検討した。対象者は、埼玉県伊奈町にて実施された小児生活習慣病予防検診を受診したすべての小学4年生（小4、1994～2010年）および中学1年生（中1、1997～2010年）10894人とした。検診の実施に際しては、あらかじめ対象者および保護者から書面による同意を得た。同検診において、身長、体重、血圧を測定した。体格は、Body Mass Indexを算出し、Centers for Disease Control and Preventionの基準に従って、「非過体重・非肥満」「過体重」「肥満」に分類した。解析は、血圧を従属変数、西暦を独立変数として直線回帰分析を行った。収縮期血圧の年次推移は、小4および中1とも、性別にかかわらず有意な減少傾向を示した。（小4男児：-0.350、女児：-0.513、中1男児：-0.434、女児：-0.473mmHg/年）（すべてのP値＜ 0.001）。体格別の解析においても、小4および中1男児では体格に関係なく収縮期血圧は減少傾向を示した。拡張期血圧は、小4では性・体格に関係なく有意な減少傾向を示したが、中1では男児の過体重群、肥満群および女児の肥満群において有意な減少傾向は認めなかった。過去17年間、小4では性・体格に関係なく血圧は減少傾向を示した。しかし、中1では、体格別に検討した場合、肥満群において血圧の年次推移に有意な傾向は認められなかった。

## INTRODUCTION

Obesity is associated with risk factors for cardiovascular disease (CVD), including hypertension, diabetes mellitus, and dyslipidemia.^1^ Blood pressure (BP) values are significantly higher among children and adolescents who are obese than among those who are not.^2^^–^^4^ Studies have tracked BP from childhood to adulthood,^5^^,^^6^ and monitoring secular trends in BP among children is important in predicting subsequent hypertension and CVD.

Current trends in BP among children have been reported from the United States,^7^^–^^11^ Africa,^12^ and other countries.^13^^–^^15^ However, most such studies compared BP at only 2^7^^–^^9^^,^^13^ or 3 time points,^10^^,^^12^^,^^14^ and the findings were inconsistent. Some studies found a tendency towards increased BP,^7^^,^^8^ whereas others observed the opposite.^13^ Therefore, assessing secular trends in BP over a longer period might be important.

BP has not been measured often among populations of Japanese children at the population level because it is not included in the annual health checks at Japanese elementary and junior high schools. Therefore, only a few epidemiologic studies have examined BP in Japanese children^16^^–^^18^ and, to our knowledge, only 1 study has analyzed secular trends in BP, BMI, and serum lipids among Japanese schoolchildren (from 1993 to 2008).^19^ In that study, the 95th, 50th, and 5th percentiles of SBP, DBP, and BMI were calculated for each year, and trends in the variables were shown in relation to calendar year. Because BP has been reported to be closely associated with overweight and obese,^2^^–^^4^^,^^18^ it could be useful to consider physique (overweight/obese vs non-overweight/obese) when observing secular trends in BP among population-based schoolchildren.

Accordingly, the present study investigated secular trends in BP using data from population-based annual screens of Japanese schoolchildren and examined the effects of sex, grade level, and physique on these trends.

## METHODS

As part of its community health services, the town of Ina in Saitama Prefecture, Japan has conducted annual health checks to prevent childhood lifestyle-related diseases since 1994, and the outcomes of this program have been reported.^18^^,^^20^^–^^26^ The present study was part of that program.

### Participants

The participants of this study comprised all fourth graders (age 9–10 years) who lived in Ina between 1994 and 2010 and all seventh graders (age 12–13 years) who lived in Ina between 1997 and 2010. Written informed consent was obtained from the parents or guardians of the children, and the Medical Ethics Committee of Showa University School of Medicine approved the study protocol.

### Data collection

We collected information on the age, sex, height, weight, and BP of each child. The annual school health examination was conducted at each school in June during 1994 to 2007 and in September during 2008 to 2010. The same examination protocol was used throughout the period from 1994 through 2010 to ensure uniformity of quality control and precision of assessment. All children wore light clothing but removed their shoes and socks, after which height and weight were measured in increments of 0.1 cm and 0.1 kg, respectively. Body mass index (BMI) was calculated as body weight (kg) divided by the square of the height (m^2^).

After the children had rested for at least 5 minutes, systolic BP (SBP) and diastolic BP (DBP) were measured in the right upper arms of seated children by well-trained nurses and medical technologists using a mercury sphygmomanometer, a stethoscope, and cuffs. The measurements were done in the school’s infirmary or in a designated room, to protect the privacy of children during the procedures. Nine-centimeter and 12-cm cuffs were used for fourth and seventh graders, respectively. A 12-cm cuff was used for fourth graders if the 9-cm cuff was too small. When SBP was 120 mm Hg or higher, or DBP was 70 mm Hg or higher, BP was measured 3 times and the third value was recorded.^18^

### Definition of physique

According to the BMI for age-weight status categories (ie, underweight, healthy weight, overweight and obese) of the Centers for Disease Control and Prevention (CDC),^27^ we classified children as non-overweight/-obese, overweight (BMI equal to or above the sex-specific 85th percentile but less than the 95th percentile of BMI for age), and obese (BMI ≥95th percentile for age). Non-overweight/-obese was regarded as a BMI less than the 85th sex-specific percentile of BMI for age.

### Statistical analysis

Data were analyzed separately according to grade level (fourth or seventh) and sex. Characteristics of boys and girls were compared using the unpaired *t* test after normality of distribution was tested for each variable. Secular trends in BP were evaluated by regression analysis; calendar year was the independent variable, and BP (SBP or DBP) was the dependent variable by sex, grade level, and physique (non-overweight/-obese, overweight, obese). The sample size for each year was adjusted using the regression analysis-weighted least-squares method.^19^ A *P* value of less than 0.05 was considered statistically significant. All data were analyzed using SPSS 16.0J (IBM, Chicago, IL, USA).

## RESULTS

Of the 10 972 children, 78 were excluded from the analysis because of refusal to participate or school absence. Thus, data from 10 894 children were analyzed. The rate of participation for the fourth and seventh graders was 99.3%. Almost all the fourth graders during 1994 to 2007 (*n* = 4333) were among the seventh graders during 1997 to 2010 (*n* = 4736).

Table 1 shows the characteristics of the participants. Among the 10 894 children, 6158 were fourth graders (3162 boys and 2996 girls) and 4736 were seventh graders (2368 boys and 2368 girls), with mean ages of 9.3 and 12.3 years, respectively. The mean SBP values for fourth-grade boys and girls were 110.9 and 110.0 mm Hg, respectively, whereas those for seventh-grade boys and girls were 110.4 and 108.3 mm Hg, respectively. The values were significantly higher in boys than in girls in both grades (fourth grade, *P* = 0.004; seventh grade, *P* < 0.001). The mean DBP values for fourth-grade boys and girls were 60.5 and 60.4 mm Hg, respectively, whereas those for seventh-grade boys and girls were 57.5 and 58.3 mm Hg, respectively. DBP did not significantly differ between sexes (*P* = 0.537) among fourth graders but was significantly higher in girls than in boys among seventh graders (*P* = 0.001).

**Table 1.** Characteristics of study participants

	Fourth graders			Seventh graders		
	
	Boys	Girls	*P*-value^a^	Boys	Girls	*P*-value^a^
	*n* = 3162	*n* = 2996		*n* = 2368	*n* = 2368	
Age (years)	9.3 (0.5)	9.3 (0.5)	0.850	12.4 (0.5)	12.3 (0.5)	0.483
Height (cm)	135.0 (5.8)	135.1 (6.4)	0.568	154.9 (8.1)	153.1 (5.8)	<0.001
Weight (kg)	31.8 (6.9)	31.0 (6.4)	<0.001	45.3 (10.2)	44.3 (8.1)	<0.001
BMI (kg/m^2^)	17.3 (2.8)	16.9 (2.5)	<0.001	18.8 (3.1)	18.8 (2.8)	0.354
SBP (mm Hg)	110.9 (11.0)	110.0 (11.5)	0.004	110.4 (11.4)	108.3 (10.5)	<0.001
DBP (mm Hg)	60.5 (9.3)	60.4 (9.4)	0.537	57.5 (8.5)	58.3 (8.3)	0.001

BMI, body mass index; SBP, systolic blood pressure; DBP, diastolic blood pressure.

Values are mean (SD).

^a^The unpaired *t*-test was used to compare characteristics between boys and girls.

Table 2 and Figure show secular trends in SBP, DBP, and BMI between 1994 and 2010. SBP was significantly associated with calendar year from 1994 through 2010 in fourth graders. Regression analysis showed decreases in SBP of 0.350 and 0.434 mm Hg/year among fourth-grade boys and girls, respectively (both *P* < 0.001). Among seventh graders, SBP negatively correlated with calendar year between 1997 and 2010, with decreases of 0.513 and 0.473 mm Hg/year among boys and girls (both *P* < 0.001). In addition, DBP and BMI were significantly negatively correlated with calendar year regardless of sex or grade level.

**Figure.**
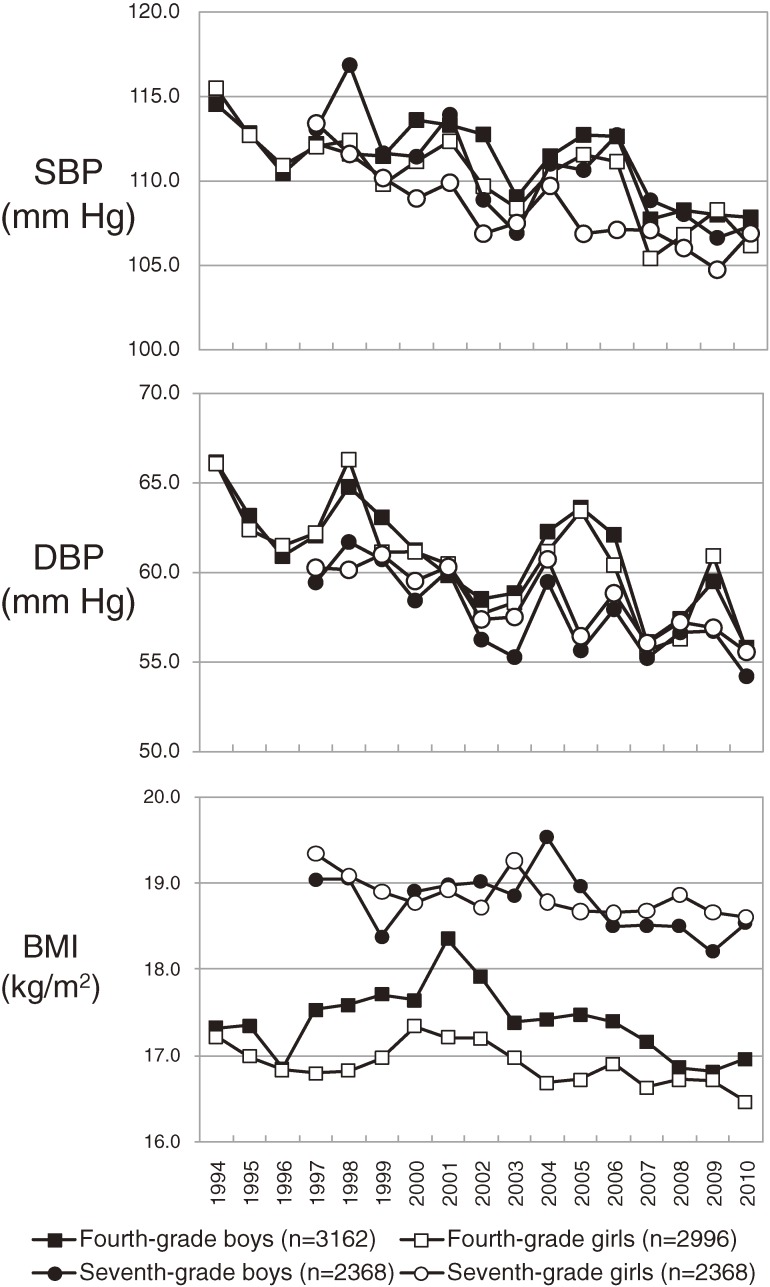
Secular trends in mean systolic blood pressure (SBP), diastolic blood pressure (DBP), and body mass index (BMI) in schoolchildren

**Table 2.** Secular trends in BMI, SBP, and DBP by sex

		Fourth graders (1994–2010)			Seventh graders (1997–2010)		
			
		*n*	Regression coefficient (95% CI)	*P*-value	*n*	Regression coefficient (95% CI)	*P*-value
BMI							
	Boys	3162	−0.039 (−0.059 to −0.020)	<0.001	2368	−0.046 (−0.076 to −0.016)	0.003
	Girls	2996	−0.030 (−0.048 to −0.013)	0.001	2368	−0.038 (−0.063 to −0.012)	0.005
SBP							
	Boys	3162	−0.350 (−0.424 to −0.276)	<0.001	2368	−0.513 (−0.619 to −0.406)	<0.001
	Girls	2996	−0.434 (−0.513 to −0.355)	<0.001	2368	−0.473 (−0.567 to −0.379)	<0.001
DBP							
	Boys	3162	−0.451 (−0.514 to −0.389)	<0.001	2368	−0.418 (−0.498 to −0.338)	<0.001
	Girls	2996	−0.436 (−0.500 to −0.372)	<0.001	2368	−0.355 (−0.430 to −0.280)	<0.001

BMI, body mass index; SBP, systolic blood pressure; DBP, diastolic blood pressure.

The independent variable was study year; the dependent variables were SBP and DBP.

Table 3 shows BP trends by physique. Both SBP and DBP were significantly associated with calendar year, regardless of sex or physique, in fourth graders, among whom SBP and DBP decreased. In contrast, SBP and DBP were negatively correlated with calendar year regardless of sex among seventh graders. However, SBP did not significantly correlate with calendar year among obese seventh-grade girls, and DBP did not significantly correlate with calendar year among overweight or obese seventh-grade boys or among obese seventh-grade girls.

**Table 3.** Secular trends in SBP and DBP by physique

		*n*	SBP		DBP	
			
			Regression coefficient (95% CI)	*P*-value	Regression coefficient (95% CI)	*P*-value
Fourth graders (1994–2010)						
Boys	non-overweight	2468	−0.324 (−0.403 to −0.245)	<0.001	−0.399 (−0.467 to −0.330)	<0.001
	overweight	404	−0.306 (−0.516 to −0.097)	0.004	−0.589 (−0.768 to −0.410)	<0.001
	obese	290	−0.288 (−0.539 to −0.038)	0.024	−0.527 (−0.750 to −0.304)	<0.001

Girls	non-overweight	2586	−0.353 (−0.434 to −0.272)	<0.001	−0.400 (−0.468 to −0.332)	<0.001
	overweight	278	−0.553 (−0.822 to −0.284)	<0.001	−0.507 (−0.729 to −0.284)	<0.001
	obese	132	−0.915 (−1.294 to −0.536)	<0.001	−0.514 (−0.817 to −0.212)	0.001

Seventh graders (1997–2010)						
Boys	non-overweight	2005	−0.455 (−0.564 to −0.345)	<0.001	−0.432 (−0.517 to −0.347)	<0.001
	overweight	219	−0.458 (−0.781 to −0.135)	0.006	−0.278 (−0.559 to 0.003)	0.052
	obese	144	−0.628 (−1.040 to −0.216)	0.003	−0.099 (−0.427 to 0.229)	0.553

Girls	non-overweight	2083	−0.438 (−0.536 to −0.340)	<0.001	−0.328 (−0.405 to −0.250)	<0.001
	overweight	217	−0.628 (−0.925 to −0.332)	<0.001	−0.489 (−0.765 to −0.214)	0.001
	obese	68	−0.330 (−0.927 to 0.267)	0.274	−0.412 (−0.912 to 0.088)	0.105

SBP, systolic blood pressure; DBP, diastolic blood pressure.

## DISCUSSION

We found a significant negative correlation between BP and calendar year regardless of sex or physique among fourth graders between 1994 and 2010 in the town of Ina in Saitama Prefecture, Japan. Moreover, BP and calendar year were negatively correlated among seventh-grade boys and girls between 1997 and 2010. These results are consistent with those of recent studies.^13^^,^^19^ Kouda et al analyzed secular trends in BP and BMI among fifth graders in Japan and found that SBP and DBP had decreased in schoolchildren during a recent 15-year period (1993–2008).^19^

In the present study, BP might have decreased due to a reduction in BMI. Raj et al reported that trends in BMI status among children were closely associated with BP in childhood.^28^ In fact, the present study found a significant negative correlation between BMI and calendar year regardless of grade level or sex. Annual health checks to prevent development of childhood lifestyle-related diseases have been conducted in Ina since 1994. This process has enhanced the awareness of parents and guardians regarding obesity and childhood lifestyle-related diseases, which might have helped reduce BMI among their children, thereby decreasing BP. However, according to the Japanese National Nutrition Survey from 1998 to 2008, change in BMI among Japanese youth was minimal among boys (from 21.2 to 21.0) and girls (from 20.6 to 20.5),^29^ but change in BMI in overweight/obese (BMI ≥25.0) children and underweight (BMI <18.5) children was more obvious in both sexes. In addition, the prevalence of overweight/obesity increased among boys, and underweight increased among girls. A previous study suggested that the increased prevalence of underweight Japanese youth offset the increase in overweight/obese youth; thus, average BMI change among Japanese youth is deceiving.^30^ Because BP is closely associated with overweight and obesity,^2^^–^^4^^,^^18^ it might be necessary to consider physique when evaluating BP trends. Therefore, we first investigated the secular trend in BP in analysis stratified by physique and in multiple regression analysis that adjusted for physique. BP was significantly negatively correlated with calendar year in multiple regression analysis (data not shown), whereas the results differed by physique in stratified analysis. Accordingly, we showed the secular trend in BP by physique and included physique in our discussion of BP trends among children.

When BP trends were analyzed by sex, grade level, and physique, BP decreased regardless of sex or physique among fourth graders. However, SBP and calendar year did not significantly correlate among obese seventh-grade girls, and DBP likewise did not significantly correlate among overweight and obese seventh-grade boys or obese seventh-grade girls. Thus, secular trends in BP differed between seventh-grade boys and girls. Such differences matter at that age because they might be associated with differences in the degree of sexual maturity, which is associated with BP level.^31^ The results from the seventh-grade children might be due to an association between BP and abdominal obesity, which might have been caused by changes in body composition.^32^ Thus, future studies should investigate percent body fat and waist circumference, in addition to the influence of physique based on BMI.

To our knowledge, this study is the first to report secular BP trends by physique (underweight, healthy weight, overweight, and obese) among population-based schoolchildren in Japan over a long period (1994–2010). Data were obtained using a consistent measurement protocol from all fourth-grade and seventh-grade children in annual health checkups. Therefore, the strength of our study is that secular trends in BP were examined with regard to physique, which differs from the methods of a similar study.^19^ However, there are a few limitations that warrant mention. First, we did not consider eating behavior, salt intake, or physical activity, all of which might contribute to BP trends. The decline in BP in the present study might have been due to a change in dietary habits, such as decreased salt intake.^33^ These factors should be considered in future studies. Second, our results might have been affected by the time of data collection; for example, heights and weights collected in September (1994–2007) could have been greater than those obtained in June (2008–2010), which might influence secular trends in BMI in the present study. However, BMI remained negatively correlated with calendar year even when we excluded data for 2008–2010 in the analysis. Finally, since the children were from only 1 Japanese town, the generalizability of the present findings might be limited. However, the height and weight of the children in Ina were similar to the national averages for Japanese children.^34^

In conclusion, BP decreased among fourth graders from Ina over the past 17 years, regardless of sex or physique. Although BP decreased among seventh graders, BP and calendar year did not significantly correlate among those who were obese. Secular trends in BP among children are predictors of future disease prevalence. The present results also suggest that physique should be considered when discussing BP trends among schoolchildren and that secular trends in BP should be assessed over longer periods of time. Monitoring secular trends in BP among schoolchildren is an important contribution to primary prevention of childhood lifestyle-related diseases, including hypertension.

## ONLINE ONLY MATERIALS

Abstract in Japanese.
