# Supplementary material for: Secular Trends in Blood Pressure Among Japanese Schoolchildren: A Population-Based Annual Survey From 1994 to 2010
Source: J Epidemiol. 2012 Sep 5;22(5):448–53. doi: 10.2188/jea.JE20110137 (PMC3798640; doi:10.2188/jea.JE20110137)
Supplement: Abstract in Japanese. [file je-22-448-s001.pdf]

日本における小児の血圧年次推移－1994～2010 年

白澤貴子[1]、落合裕隆[1]、西村理明[2]、森本彩[2]、島田直樹[1]、大津忠弘[1]、星野祐美[1]、田嶼尚子[3]、小風暁[1]

[1]昭和大学医学部公衆衛生学部門、[2]東京慈恵会医科大学糖尿病・代謝・内分泌内科
[3]東京慈恵会医科大学

小児期の血圧の年次推移を観察することは、将来の高血圧や心血管疾患発症の動向を予測する際に重要である。そこで本研究は、日本人学童・生徒における血圧の年次推移について体格別に検討した。対象者は、埼玉県伊奈町にて実施された小児生活習慣病予防検診を受診したすべての小学 4 年生（小 4、1994～2010 年）および中学 1 年生（中 1、1997～2010 年）10894 人とした。検診の実施に際しては、あらかじめ対象者および保護者から書面による同意を得た。同検診において、身長、体重、血圧を測定した。体格は、Body Mass Index を算出し、Centers for Disease Control and Prevention の基準に従って、「非過体重・非肥満」「過体重」「肥満」に分類した。解析は、血圧を従属変数、西暦を独立変数として直線回帰分析を行った。収縮期血圧の年次推移は、小 4 および中 1 とも、性別にかかわらず有意な減少傾向を示した。（小 4 男児：-0.350、女児：-0.513、中 1 男児：-0.434、女児：-0.473mmHg/年）（すべての P 値＜ 0.001）。体格別の解析においても、小 4 および中 1 男児では体格に関係なく収縮期血圧は減少傾向を示した。拡張期血圧は、小 4 では性・体格に関係なく有意な減少傾向を示したが、中 1 では男児の過体重群、肥満群および女児の肥満群において有意な減少傾向は認めなかった。過去 17 年間、小 4 では性・体格に関係なく血圧は減少傾向を示した。しかし、中 1 では、体格別に検討した場合、肥満群において血圧の年次推移に有意な傾向は認められなかった。

キーワード：血圧、Body Mass Index、小児、年次推移
